# Effects of Conformism on the Cultural Evolution of Social Behaviour

**DOI:** 10.1371/journal.pone.0068153

**Published:** 2013-07-10

**Authors:** Lucas Molleman, Ido Pen, Franz J. Weissing

**Affiliations:** 1 PhD student, Theoretical Biology Group, Centre for Ecological and Evolutionary Studies, University of Groningen, Groningen, The Netherlands; 2 Professor of Theoretical Biology, Theoretical Biology Group, Centre for Ecological and Evolutionary Studies, University of Groningen, Groningen, The Netherlands; 3 Professor of Theoretical Biology Theoretical Biology Group, Centre for Ecological and Evolutionary Studies, University of Groningen, Groningen, The Netherlands; Universidad Carlos III de Madrid, Spain

## Abstract

Models of cultural evolution study how the distribution of cultural traits changes over time. The dynamics of cultural evolution strongly depends on the way these traits are transmitted between individuals by social learning. Two prominent forms of social learning are payoff-based learning (imitating others that have higher payoffs) and conformist learning (imitating locally common behaviours). How payoff-based and conformist learning affect the cultural evolution of cooperation is currently a matter of lively debate, but few studies systematically analyse the interplay of these forms of social learning. Here we perform such a study by investigating how the interaction of payoff-based and conformist learning affects the outcome of cultural evolution in three social contexts. First, we develop a simple argument that provides insights into how the outcome of cultural evolution will change when more and more conformist learning is added to payoff-based learning. In a social dilemma (*e.g.* a Prisoner’s Dilemma), conformism can turn cooperation into a stable equilibrium; in an evasion game (*e.g.* a Hawk-Dove game or a Snowdrift game) conformism tends to destabilize the polymorphic equilibrium; and in a coordination game (*e.g.* a Stag Hunt game), conformism changes the basin of attraction of the two equilibria. Second, we analyse a stochastic event-based model, revealing that conformism increases the speed of cultural evolution towards pure equilibria. Individual-based simulations as well as the analysis of the diffusion approximation of the stochastic model by and large confirm our findings. Third, we investigate the effect of an increasing degree of conformism on cultural group selection in a group-structured population. We conclude that, in contrast to statements in the literature, conformism hinders rather than promotes the evolution of cooperation.

## Introduction

Social learning enables humans to survive in a broad array of different habitats across the planet. By learning from their peers, individuals can rapidly acquire adaptive information about which behaviour is optimal under a variety of environmental conditions. Models of cultural evolution use insights from theories of genetic evolution to study how cultural variants, such as ideas and beliefs, spread through populations of individuals by social learning. Social learning based on imitating the behaviour of successful individuals can lead to an evolutionary dynamic similar to the spread of alleles under natural selection, whereas learning by adopting behaviours from others more randomly leads to a process resembling genetic drift.

Models of cultural evolution have to be adapted to the specific mechanisms by which cultural traits transmit between individuals. Traits can be transmitted not only vertically from parents to offspring, but in a range of different ways. For instance, traditional hunters may learn from their parents a social norm to share hunting revenues, and may learn the optimal design of an arrow from their fellow hunters. How humans learn from each other is a topic of extensive theoretical and empirical research (for a recent overview see [1]), and various specific forms of social learning (termed ‘social learning strategies’ [2] or ‘learning biases’ [3]) have been studied as to how they affect the spread of cultural traits through populations. Two forms of social learning received particular attention: conformism and payoff-based learning.

When individuals can evaluate the payoffs of the behavioural strategies of others, the preferential imitation of high-payoff individuals can lead to the rapid spread of adaptive behaviours in a population [4]. However, such payoff-based learning is not always feasible. Getting insights in the payoffs received by others is not always straightforward, especially for newcomers in a population. In cases like this, imitating the majority (conformism) can be a good alternative form of social learning, in particular if the success of cultural traits strongly depends on the local circumstances [3], [5].

The role of conformism in cultural evolution has recently become the matter of considerable debate. In the context of a social dilemma, payoff-based learning will tend to inhibit the spread of cooperation because defectors obtain higher payoffs by reaping the benefits of cooperation without paying the costs. Theory suggest that when payoff-based learning is complemented by other forms of social learning, the dynamics of cultural evolution can be strongly affected. For instance, adding random learning to payoff-based learning can facilitate the rapid solution of coordination problems [6], and conformism can stabilise cooperative equilibria under specific conditions [3], [7]–[14]. Moreover, conformism can homogenise groups internally, thereby augmenting the relative amount of variation *between* groups [14]. This decreases the scope for selection within groups (*i.e.* payoff-based transmission disfavouring cooperation), and increases the potential role of ‘cultural group selection’. In group-structured populations, cooperation can spread when groups of cooperators have some advantage over groups of defectors. This advantage can manifest itself in a number of different ways; cooperative groups may send out more migrants, grow to larger sizes, or replace other groups (*e.g.* [8], [15]–[17]). Selection at the group level may also occur when individuals occasionally learn from members of other groups that perform better[4], [18]–[20]. Through such a process, cooperation can be promoted since individuals in cooperative groups have higher payoffs than individuals in groups of non-cooperators.

Experiments from psychology and behavioural economics suggest that humans indeed use both conformist and payoff-based learning in determining their behaviour [21]–[24]. When individuals are allowed to use both conformist and payoff-based learning, experimental evidence suggests that cultural traits can spread through a *mixture* of these two forms of social learning [23], [25], [26]. This raises the question of how the interplay of conformist and payoff-based learning affects the spread of cultural traits through a population.

First, we develop a simple argument to delineate how the direction of cultural change is affected by the relative rate of conformist and payoff-based learning. This will give us an intuitive insight in the effects of conformism in various contexts of social interaction. Second, we construct stochastic models that allow us to follow the spread of culturally transmitted behaviours in the course of time in a finite population. These models allow us to quantify how the relative degree of conformism (as opposed to payoff-based learning) affects the success of social strategies in reaching fixation. Third, we examine how cooperation can spread in a group-structured population by means of cultural group selection. With this model, we investigate whether conformism tends to promote – as often claimed in the literature – or hamper the spread of cooperation in populations that are structured into groups of finite size.

## Analysis and Results

### 1. Model Structure

We consider a population in which individuals are involved in social interactions. Individuals have a culturally acquired strategy that determines their behaviour in these interactions. We consider two variants of this behaviour (*A* or *B*). An individual has the inclination of playing either *A* or *B*, but this inclination can change over the course of time due to social learning. Learning is either based on payoffs (individuals tend to imitate successful individuals) or on conformism (individuals tend to imitate the majority of the population). The relative frequency *γ* of these two forms of social learning is the key parameter of interest. The value of *γ* ranges from 0 to 1. If *γ* = 0, all learning is payoff-based; if *γ* = 1, all learning is based on conformism. If 0< *γ* <1, individuals use a mixture of these two forms of social learning. We assume that all individuals use the same mixture of conformist and payoff-based learning.

Individuals acquire payoffs by social interaction with others in their group. Payoffs depend linearly on the frequency *p* of *A*-individuals (and 1–*p* of *B*-individuals), and are calculated using the payoff matrix 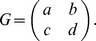 The payoff of *A*-individuals is
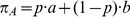
(1a)and the payoff of *B*-individuals is



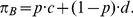
(1b)There are three strategically different classes of games with two pure strategies, and we consider the evolutionary dynamics in each of these ‘interaction contexts’. In the first class of games, one of the pure strategies (say *B*) is dominant over the other: 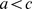 and 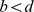. In the special case where 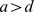, this is a *social dilemma*. Collective interests are opposed to individual interests: when all individuals exhibit behaviour *A* (‘cooperate’), payoffs are higher than when all individuals exhibit behaviour *B* (‘defect’). Individually, however, *B* yields higher payoffs than *A*, irrespective of what others are doing. Second, we consider the class of *coordination games*, which are characterized by 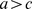 and 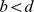. In this case, the payoff of a pure strategy increases with the number of individuals using this strategy. In a coordination game, both pure strategies are Nash equilibrium strategies. In addition, there is a (dynamically unstable) mixed-strategy equilibrium at
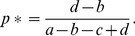
(2)


Third, we consider the class of *evasion games*, where 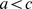 and 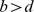. Now the relative payoff of each pure strategy decreases with the frequency of this strategy in the population. In an evasion game, none of the pure strategies is a Nash equilibrium; instead an evasion game has a unique mixed-strategy equilibrium, which is given by eq. (2). The Hawk-Dove game and the Snowdrift game are prominent examples of evasion games.

### 2. Representation of Conformism by a Coordination Game

Before studying the dynamics of cultural evolution in finite populations, we aim to get some intuition on how conformism might change the direction of cultural evolution. To this end, we represent conformism-based learning by payoff-based learning in the coordination game with payoff matrix 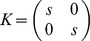. In this coordination game, the payoff to each pure strategy is proportional to its frequency in the population: 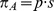 and 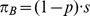, where *s* is positive. Accordingly, the pure strategy with highest frequency is favoured by payoff-based learning, just as it is in case of conformism-based learning. Based on these considerations, a mixture of payoff-based learning (characterised by matrix *G*) and conformist learning (characterised by matrix *K*) can be described by the combined matrix
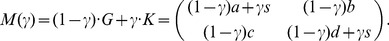
(3)


It is now straightforward to characterise the expected direction of cultural change as a function of our key parameter *γ* by determining the Nash equilibrium strategies of the matrix game *M*(*γ*). This can be done with standard methods [27]: Pure strategy *A* is a Nash equilibrium if 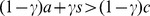 or equivalently 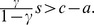 Similarly, pure strategy *B* is a Nash equilibrium when 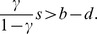 Both inequalities are more easily satisfied for larger values of *s* or *γ* and will always hold if *γ* approaches 1. When both inequalities are reversed, *M*(*γ*) has a (dynamically stable) mixed Nash equilibrium at
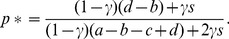
(4)


**Figure 1:**
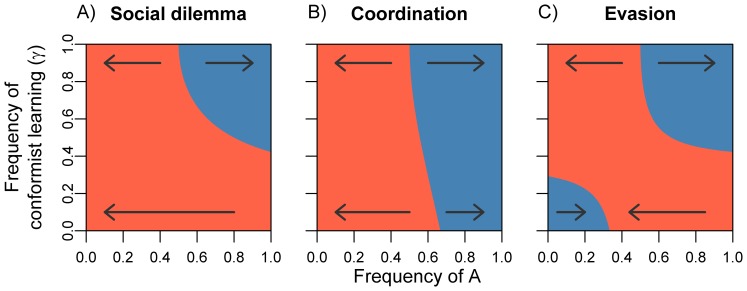
Effect of conformism on the dynamics of cultural evolution when conformism-based learning is represented by a coordination game. In three interaction contexts (social dilemma, coordination game, evasion game), colours and arrows indicate the expected change in the frequency of behavioural strategy *A* for a given value of *γ*, the relative frequency of conformism-based learning. In blue regions (arrows to the right), *A* tends to increase; in red regions (arrows to the left), *A* tends to decrease. When all learning is conformism-based (*γ* = 1), cultural evolution will either lead to the fixation of *A* or to the fixation of *B*, depending on which strategy was initially most frequent in the population. When all learning is payoff-based (*γ* = 0), strategy *A* (‘cooperation’) will disappear in the social dilemma; either *A* or *B* will go to fixation in the coordination game; and *A* and *B* will stably coexist in the evasion game. Changing *γ* from 0 to 1 leads to a smooth transition between these two scenarios. Parameters settings: *s* = 0.2; payoff matrices: social dilemma 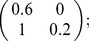 coordination game 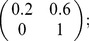 and evasion game 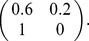

For each of the three interaction contexts, Figure 1 illustrates how the dynamics of cultural evolution (increase or decrease in the frequency of pure strategy *A*) changes with the frequency of conformist learning *γ*. First, consider the extreme case 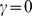, at the bottom of the three panels of Figure 1. Here, all learning occurs on the basis of the payoffs in matrix *G*. In the *social dilemma*, *A* (cooperate) is disfavoured by payoff-based learning, and cultural evolution will lead to a decrease of the frequency of *A* (Figure 1A, bottom arrow to the left) and convergence to the sole Nash equilibrium 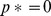. The *coordination game* has two pure-strategy Nash equilibria (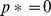 and 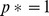) that are separated by the dynamically unstable mixed-strategy Nash equilibrium (eq. 2). The two arrows at the bottom of Figure 1B indicate that cultural evolution will either lead to the fixation of *A* or to the fixation of *B*, and that the outcome depends on initial conditions. In the *evasion game*, the arrows at the bottom of Figure 1C indicate that the system will converge to the mixed-strategy Nash equilibrium (eq. 2), where *A* and *B* stably coexist. Next consider the other extreme 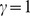, where all learning is conformism based, i.e. governed by matrix *K* (top of the three panels in Figure 1). Now the expected direction of change is identical for each of the three interaction contexts: since *K* is a coordination game, the two pure strategies are Nash equilibria and cultural evolution will either lead to the fixation of *A* or to the fixation of *B*, depending on initial conditions (top arrows in all three panels). Due to conformist learning, the strategy that is initially more abundant is most likely to spread to fixation.

For intermediate frequencies of conformism 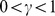, we observe a gradual shift between the two extreme cases 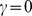 and 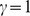. In the *social dilemma* (Figure 1A), cooperation becomes a stable equilibrium as soon as conformism is sufficiently strong (i.e. if 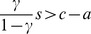): when a group mainly consists of cooperators, conformist learning leads to the maintenance and fixation of this most abundant strategy, despite of its payoff disadvantage. In the *coordination game* (Figure 1B), the direction of change remains qualitatively unchanged, but the unstable Nash equilibrium separating the basins of attraction shifts from eq. (2) to p* = 0.5, the mixed-strategy Nash equilibrium of the coordination game *K*. In the *evasion game* (Figure 1C), *A* and *B* coexist at equilibrium when learning is mainly based on payoffs (small *γ*). High frequencies of conformism *γ* decrease the scope for this coexistence. When conformism occurs at a sufficiently high frequency (moving towards the top of the panel), the direction of expected change is reversed, and the two monomorphic equilibria become stable.

### 3. A Stochastic Model for Cultural Evolution in Finite Populations

The approach taken above provides an intuitive understanding of the role of conformism in various types of interaction contexts. However, it is not clear whether, and to what extent, the features of conformism-based learning are captured by a coordination game. We therefore developed a dynamic model for cultural evolution where conformism is represented in a more mechanistic way. Cultural evolution takes place in finite (and often small) populations, where chance events may play an important role. Accordingly, we consider a stochastic model for cultural evolution in a finite population of fixed size *n*. Our model is event-based, where an ‘event’ corresponds to a potential change in strategy by one population member. In each time step (i.e. when an event occurs) two individuals are chosen at random from the population, and one of them is allowed to update its behavioural strategy (*A* or *B*) by learning from the other. Updating occurs either through conformist or payoff-based learning. For each state of the population (i.e. each possible frequency of *A*-strategists), we calculate the probability that an *A*-individual switches to *B*, and that a *B*-individual switches to *A*. At the population level, each such switch corresponds to a decrease or to an increase of the number of *A*-individuals by one. Since we neglect the spontaneous emergence of *A*- or *B*-strategists (the cultural equivalent of genetic mutations), the stochastic process will eventually lead to an ‘absorbing state’, where all individuals have adopted either of the two strategies. To assess the effects of conformist learning on the outcome of cultural evolution, we evaluate how our key parameter *γ* affects the fixation probability and waiting time to fixation for each of the two behavioural strategies [28].

Let *i* be the number of *A*-strategists in the population, and let 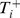 and 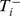 denote the probability of gaining resp. losing one *A*-individual. We model the switching dynamics by using pairwise comparison (cf. [7]). When an event occurs, two individuals are chosen at random from the population. A change in strategy can only take place when these two individuals have opposite strategies, which occurs with probability 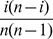. In state *i*, switching from *A* to *B* (probability 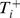) and from *B* to *A* (probability 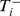) occurs with probabilities:
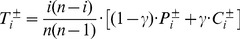
(5)where *C* and *P* denote the probabilities of switching due to conformist and payoff-based learning, respectively. For both forms of social learning, we specify the switching probabilities as a logistic function of the differences in payoffs (in case of payoff-based learning) or frequencies (in case of conformist learning) between strategies *A* and *B*. In state *i*, payoff-based switching from *B* to *A* (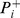), and from *A* to *B* (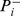) occurs with probabilities



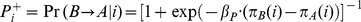
(6a)

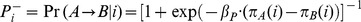
(6b)where 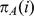 and 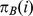 (see eq. (1)) refer to the payoffs of strategies *A* and *B* in state *i*, respectively. Parameter *β_P_* quantifies the strength and direction of the relation between the payoff difference and the probability of switching. When *β_P_* = 0, payoff-based learning is not biased in any particular direction, and is expected to lead to dynamics similar to genetic drift. When *β_P_* is large, payoff-based learning is strongly biased, favouring the spread of strategies with the highest payoff (see Figure S1 for an illustration).

Conformist learning is represented in a similar way. In state *i,* conformist switching occurs with probabilities
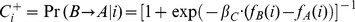
(7a)

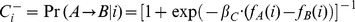
(7b)where 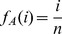 and 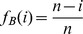 refer to the relative frequencies of strategies *A* and *B*, respectively. Parameter *β_C_* quantifies the strength and direction of frequency-based social learning. When *β_C_* = 0, such learning is not biased in any particular direction; when *β_C_* is large, individuals are strongly inclined to adopt the more frequent strategy in the population (see Figure S1).

Now we have specified 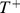 and 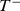 for all states *i* of the population, we can use standard methods [28] to calculate fixation probabilities φ and waiting times to fixation, for various initial abundances of strategy *A* and *B*, as a function of the relative frequency of conformism *γ*. In File S1, section 2, we show how explicit equations for the fixation probabilities can be derived on the basis of a diffusion approximation of the stochastic model.

**Figure 2:**
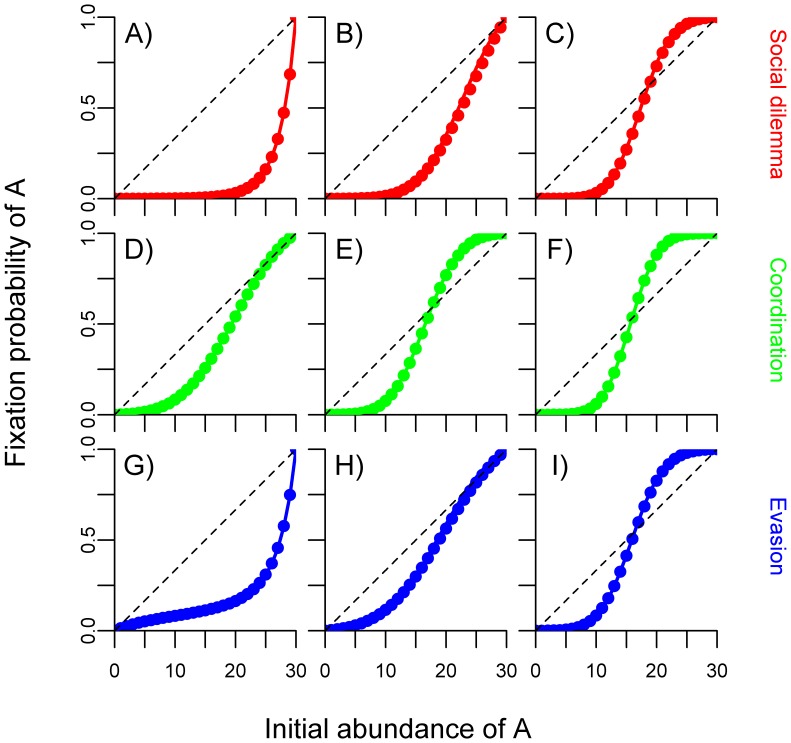
Effects of conformism on the outcome of cultural evolution in a small group. Each panel shows the fixation probability of strategy *A* as a function of its initial abundance (*k*) in a group of *n* = 30 individuals. Columns of panels correspond to three different frequencies *γ* of conformism. Symbols represent fixation probabilities from the exact stochastic model, and lines represent a diffusion approximation to these fixation probabilities (see File S1, section 2). The diagonal dashed lines indicate the fixation probability of a strategy under random drift (φ*_k_* = *k*/*n*). Parameter settings: *β_P_* = 1 and *β_C_* = 2; payoff matrices of the games as in Figure 1.

We assess the influence of the social learning rules on the outcome of cultural evolution by comparing the fixation probabilities φ*_k_* of *A* for a given initial abundance *k* of this strategy to that of a ‘neutral’ process, where all switching occurs randomly. It is well known that in the latter case φ*_k_* equals the initial frequency *k*/*n* of *A* [28]. Panels A to C in Figure 2 illustrate the effects of conformism on the fixation probability of *A* (cooperate) in a *social dilemma*. When all switching occurs on the basis of payoffs (*γ* = 0; 2A), cooperation is always disfavoured. Accordingly, fixation of *A* is very unlikely unless the initial abundance of *A* is relatively large. In fact, all fixation probabilities (red dots) are below the diagonal, indicating that fixation of *A* is for all values of *k* less likely than ‘neutral’ updating. If social learning is partly based on conformism (*γ* = 0.25, 2B; *γ* = 0.5, 2C), the fixation curve becomes more S-shaped. Strategy *A* is increasingly likely to fixate when its initial abundance is high, which agrees with our earlier findings (Figure 1A).

In the *coordination game* without conformism (Figure 2D), payoff differences near the *A* equilibrium are relatively small. This means that switching from *A* to *B* can frequently occur, despite the fact that strategy *B* yields lower payoffs. When the state of the group is close to the unstable equilibrium, such stochastic events can tip the group into the basin of attraction of *B*. As conformism increases in frequency, the pure *A* equilibrium tends to be more stable; the fixation probability rises above the diagonal *k*/*n* line when the initial abundance of A is high (Figure 2E, F). This finding is again in line with our earlier results (Figure 1B).

In the *evasion game*, payoff-based learning tends to favour the spread of rare strategies. When conformist learning is absent (*γ* = 0, Figure 2G), fixation of *B* is often more likely. The polymorphic equilibrium is located at *n*/3, and a group is expected to spend most of the time close to this equilibrium. Since this state is closer to the absorbing state where all individuals play strategy *B* (relative to the other absorbing state, where all individuals play strategy *A*), stochastic events will more likely lead to fixation of *B* rather than fixation of *A*. When conformist learning occurs at higher frequencies (Figure 2H, I), cultural evolution tends to lead to fixation of the strategy that was more abundant initially; again, this is in line with our earlier findings (Figure 1C).

**Figure 3:**
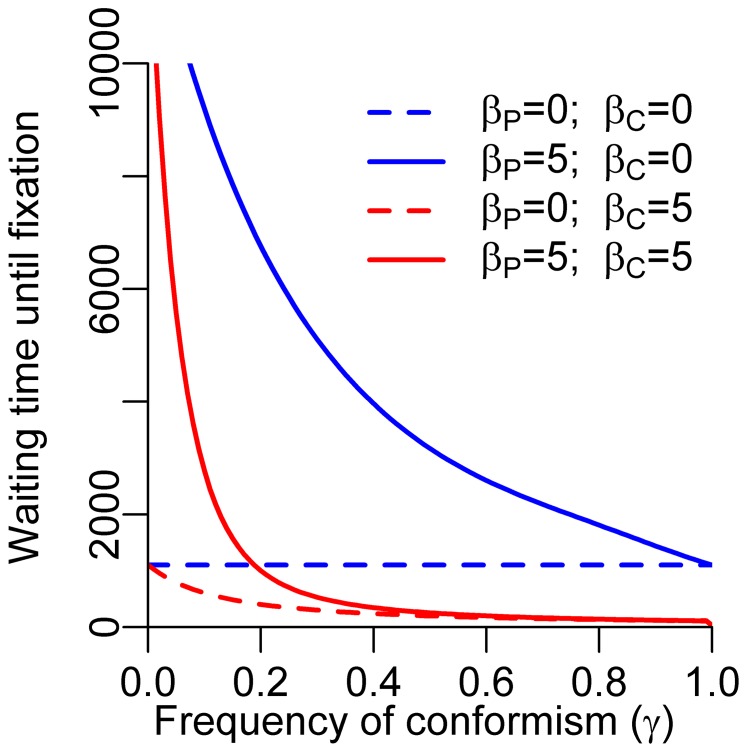
Effect of conformism on the persistence of behavioural polymorphism in an evasion game. Lines represent the expected number of updating events (i.e. the ‘waiting time’) until a group fixates in either *A* or *B*, as a function of the frequency of conformist updating *γ*. The dashed blue line indicates waiting times when all updating occurs randomly (*β_P_* = 0; *β_C_* = 0). The dashed red line reflects waiting times when conformism occurs at rate *γ*, and payoff-based learning is absent (*β_P_* = 0; *β_C_* = 1; random updating occurs at frequency 1–*γ*). The solid blue line reflects waiting times when payoff-based learning occurs at frequency 1–*γ*, and conformism is absent (*β_P_* = 1; *β_C_* = 0; random updating occurs at frequency *γ*). The solid red line represents waiting times in the full model, where payoff-based learning is complemented by conformism at rate *γ* (*β_P_* = 1; *β_C_* = 1). Groups were of size *n* = 30 and initialised at the coexistence equilibrium *p* = p* (10 *A*-individuals). Payoff matrix as in Figure 1.

Conformism also affects the time it takes until a strategy fixates in the *evasion game* (Figure 3). In an evasion game, each behavioural strategy has higher payoff when rare. Accordingly, payoff-based learning causes a group to spend a lot of time in polymorphic states before it reaches one of the absorbing states. Increasing the frequency *γ* of conformist learning has two effects: first, the frequency of payoff-based switching decreases, which hampers the spread of rare strategies, thereby destabilising the coexistence equilibrium. Second, conformist switching accelerates fixation, because individuals preferentially adopt common strategies.

By breaking down polymorphism in the evasion game, conformism affects the average payoffs of the group members. A simple calculation shows that the average payoff at the mixed-strategy Nash equilibrium (eq. 2) of an evasion game is given by 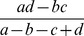. This can be considerably higher or lower than the payoff *a* in case of fixation of behavioural strategy *A* or the payoff *d* in case of fixation of *B*.

### 4. Evolution of Cooperation by Cultural Group Selection

Finally, we consider a multilevel scenario in which a metapopulation is subdivided into *m* groups of size *n*. Within groups, individuals face a social dilemma. As described above, payoff-based learning within groups tends to disfavour cooperation. Once in a while, individuals from two *different* groups are paired for updating by comparing their payoffs. This reflects a scenario where individuals occasionally copy behaviours from groups that are performing well. It might be that individuals from other groups are considered to be healthier, or have more wealth. We assume that conformism does not play a role in between-group updating (*i.e.* conformism is a strictly *local* social learning rule, allowing to cope with local conditions). Further, in our model, updating outside the group occurs at a much lower rate than within-group updating: in between two outside-group updating events. This implies that when a new strategy is newly introduced into a group, this strategy will either have gone locally extinct, or reached fixation before the next between-group event occurs. This ‘separation of time scales’ allows us to calculate the probability of fixation of a ‘cooperative’ *A* strategy in the metapopulation, by tracking the number of groups in the cooperative state. In each time step of this group level process, the abundance of cooperator groups can go up by one or go down by one, or can stay the same.

When two individuals from different groups are chosen from the population, switching probabilities are defined analogously to updating within groups. Switching probabilities depend on the payoff difference between groups where cooperation is fixated and groups where defection is fixated. This payoff difference is given by *a – d*. For this between-group process, we again use logistic functions to specify the relationship between payoff differences and the probability that one defector switches to cooperation 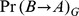 and the probability that one cooperator switches to defection 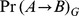.
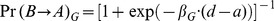
(8a)

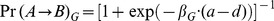
(8b)


Parameter *β_G_* specifies the relation between the payoff difference of the members of the two different groups, and the probability of switching. Since payoffs are higher in cooperative groups than in defector groups (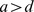), this process is expected to lead to the spread of cooperation between groups. Let *j* be the number of groups at the cooperative state, and let *m*–*j* be the number of groups at the defector state. At state *j*, the probability of gaining (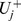) or losing (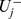) a cooperative group (by one defector taking over a cooperative group, or vice versa, by one cooperator taking over a defector group) can then be written as
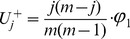
(9a)

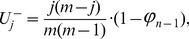
(9b)where 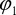 refers to the fixation probability of a single cooperator in a group of defectors, and 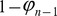 reflects the fixation probability of a single defector in a group of cooperators (which is complementary to the fixation probability of cooperation starting from state *n*–1). Using the same techniques as before, we can calculate the probability 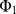 that eventually all groups have reached the cooperative state, given that we start out with 1 group of cooperators. The product 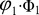 then denotes the probability that cooperation reaches fixation in the metapopulation, given that we start out with one individual with the cooperative strategy.

**Figure 4:**
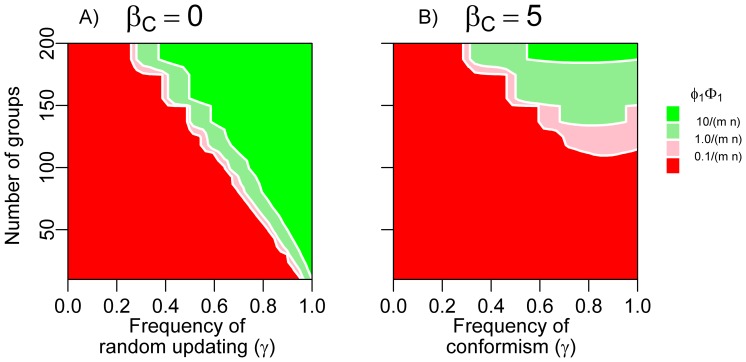
Effect of conformism on the evolution of cooperation by cultural group selection. Panels show fixation probability of cooperation in a group-structured population where initially only a single individual cooperates. Within groups, payoff-based learning favours defection, but cooperation can spread between groups by occasional learning outside of the local group, because individuals in cooperative groups have higher payoffs. The number of groups *m* was varied between 10 and 200, in steps of 5, holding the metapopulation size constant at *m n* = 1000. The frequency of conformist updating was varied between 0 and 1, in steps of 0.01. In (A), *β_C_* is equal to zero, corresponding to random updating, while the conformism-based updating has a strong effect (*β_C_* = 1) in (B). Hence, panel (A) should mainly be viewed as a reference for panel (B). We used an interpolation procedure (using the R-package ‘akima’) to smoothen the plots. Colours facilitate comparison to the fixation probability of a selectively neutral mutant 1/(*m*·*n*). The payoff matrix coincides with that of the social dilemma game in Figure 1.

Figure 4 gives an overview of the fixation probability of cooperation in case of a single cooperator in a population structured in *m* groups of fixed size *n*, under varying population structures and varying frequencies of conformism *γ*. When looking at Figure 4B only, one might conclude that conformism has a favourable effect on the cultural evolution of cooperation, since the fixation probability of cooperation tends to increase with *γ*. In our view, however, this conclusion would be misleading. The increasing scope for cooperation is not caused by conformism per se, but rather by the associated decrease in frequency of payoff-based learning. Decreased frequencies of payoff-based learning weaken selection against cooperation within the group. To assess the net effects of conformism, one therefore has to compare the results of Figure 4B with a benchmark that takes this weakening of payoff-based learning into consideration. This benchmark is presented in Figure 4A where, with probability *γ*, individuals are imitating at random.

When the frequency of random switching within groups (as opposed to payoff-based switching) increases, the scope for cooperation increases (Fig. 4A, going from the left to the right in the panel); within groups, the selection against cooperation is weakened, whereas selection between groups is kept constant (see [29] for a general analysis of how weakening within-group selection can affect the scope for cooperation in group-structured populations). Cooperation is favoured most, when the metapopulation is structured into many small groups (Fig 4A going from the bottom to the top of the panels). When groups are small, the probability that a single cooperative strategy reaches fixation – in spite of payoff-based learning disfavouring this strategy – is relatively large. Once such fixation has happened, cooperation can spread to other groups.

Comparing Figures 4A and 4B allows us to evaluate the net effects of conformism on the cultural evolution of cooperation by group selection. It is obvious that conformism hinders rather than favours the evolution of cooperation: in the whole parameter range, cooperation spreads more easily to fixation when conformism-based updating is random (*β_C_* = 0) than when it has a strong effect (*β_C_* = 5). Conformism hinders the evolution of cooperation because cooperation cannot gain a foothold in new groups: whenever a cooperator is introduced in a group of defectors, both conformist and payoff-based learning press an individual to switch back to defection.

## Discussion

By means of a simple argument (where conformism was approximated by a coordination game), we have shown that the effect of conformism on cultural evolution strongly depends on the interaction context. In case of a coordination game, conformism merely affects the basins of attraction of the two pure-strategy equilibria; in case of a social dilemma, conformism can turn cooperation into a stable Nash equilibrium that coexists with an equilibrium corresponding to pure defection; and in case of an evasion game (such as a Hawk-Dove game or a Snowdrift game), conformism can destroy a polymorphic equilibrium and induce evolution to a pure-strategy state. These conclusions were confirmed by a more mechanistic model for cultural evolution in a finite population. Including cultural group selection in this model strongly suggests that conformism tends to hinder, rather than promote the cultural evolution of cooperation by group selection.

Our approach using two-by-two matrix games with pure strategies is mathematically convenient, allowing to evaluate the success of strategies under a range of conditions in a fast and fairly straightforward manner. Also, this approach allows for deriving a diffusion approximation of the stochastic process, leading to a closed-form expression for fixation probabilities of a strategy under any mixture of payoff-based and conformist learning (see section 2 of File S1). Individual-based simulations in which switching between *A* and *B* is prone to errors – whose magnitudes are inversely related to *β_P_* and *β_C_* – lead to very similar outcomes in terms of fixation probabilities (not shown). This suggests that, despite the simplicity of our model, our findings are robust with respect to the way in which stochastic effects are introduced in the switching dynamics.

Our analysis is, however, restricted to the situation where each individual can only adopt a pure strategy. It remains unclear how conformism would influence cultural evolution when individuals are characterised by a mixed strategy, which specifies a probability distribution over the pure strategies. If such probabilistic tendencies could be transmitted between individuals by social learning, the dynamics of cultural evolution could be rather different from the scenario considered here (see [30] for an example). For instance, in an evasion game, all individuals could fixate on the same mixed strategy 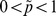, supporting a polymorphism where individuals make use of both pure strategies in a probabilistic fashion. In contrast to the findings from our analysis using two pure behaviours, conformism would be unlikely to destabilise such a behavioural polymorphism. Note, however, that the transmission of strategies by social learning depends on the degree to which individuals can evaluate the strategies of their peers. Whereas individuals might be able to evaluate the pure strategies of their peers (and possibly imitate them accordingly), it is not obvious that more complex (mixed) strategies readily transmit between individuals.

Furthermore, we assume that individuals in the population all use the same social learning strategy. Decision making experiments show that individuals tend to vary considerably in their social learning strategies (*e.g.* in the degree in which individuals learn based on payoffs; [23]). Such individual variation in social learning strategies can affect the course of cultural evolution. To see this, consider a group in which some individuals typically learn based on payoffs and others learn based on conformism. This group may reach a stable behavioural polymorphism in an evasion game: when conformists all perform a common strategy, payoff-based learners can anticipate to that by adopting the strategy that is rare. Such an emerging differentiation, in which conformist learners perform one behaviour and payoff-based learners perform another behaviour, cannot be attained by groups that are homogeneous with respect to their social learning strategies. In our model, individuals use a mixture of conformist and payoff-based learning. As a consequence, a behavioural polymorphism is destabilised by a number of consecutive conformist learning events, potentially reducing the average payoffs of individuals in a population.

Human social learning comes in many different forms, and payoff-based and conformist learning only represent those forms that have received most attention in the social learning literature. Our analysis does not account for how other relevant forms of social learning – such as following a leader or a teacher – would affect the spread of behaviours within groups (see [31] for how leadership can affect the cultural evolution of cooperation). Also, the mechanism that spreads behaviours between groups considered our model, is only one way that this group-level mechanism might work [15]. Alternative scenarios in which groups of cooperators grow faster and split up when reaching a certain size [17] are likely to lead to different outcomes of cultural evolution. Such alternative forms of group-level selection can have different consequences for evolutionary dynamics, and can interact with within-group social learning in different ways. A more specific simulation study [32], considering other population structures and different forms and social learning and group selection, arrives at a similar conclusion: conformism can promote the cultural evolution of cooperation by group selection when groups can replace other groups, whereas cooperation cannot evolve when cooperators have to spread singly from group to group by a process of ‘infection’.

Our study leads to the conclusion that conformism has only a marginal effect in the context of coordination games, that it tends to erode the polymorphic equilibrium in evasion games, and that it does not favour cooperation in a social dilemma. In other words, our evaluation of the role of conformism is considerably less favourable than the opinion of other scholars of cultural evolution [3], [8], [11], [14], [15]. To put our conclusion into context, we would like to stress that our analysis did not consider potential intrinsic benefits of conformism. Two such benefits may be relevant in a variety of situations. First, conformism could homogenise groups with respect to a diversity of norms and habits, potentially increasing social cohesion and facilitating the establishment of trust, thus making it easier to resolve internal conflicts and to get cooperation off the ground. Second, conformism could be a beneficial strategy in environments with considerable spatial variation in payoffs and/or behavioural norms. In a situation like that, newcomers in a local group or environment could profit from imitating local habits, thus quickly adopting locally superior strategies or adapting to local behavioural equilibria. More sophisticated models accounting for spatial variation and the mechanisms underlying decision making in groups might therefore arrive at a more positive judgement of the role of conformism for the cultural evolution of social behaviour.

## Supporting Information

Figure S1 Illustration of the logistic function for three values of *β*.
(TIFF)

File S1 In the first part of this supplement, we provide a graphical illustration of the logistic functions used in our stochastic model to translate information on payoff or frequency differences into the probability of switching to another strategy. In the second part, we derive a diffusion approximation of our stochastic model that yields, by good approximation, analytical expressions for the fixation probability of a strategy in a finite population as a function of the strategy’s initial frequency.
(DOCX)
