# Supplementary material for: Effects of Conformism on the Cultural Evolution of Social Behaviour
Source: PLoS One. 2013 Jul 10;8(7):e68153. doi: 10.1371/journal.pone.0068153 (PMC3707918; doi:10.1371/journal.pone.0068153)
Supplement: File S1 — In the first part of this supplement, we provide a graphical illustration of the logistic functions used in our stochastic model to translate information on payoff or frequency differences into the probability of switching to another strategy. In the second part, we derive a diffusion approximation of our stochastic model that yields, by good approximation, analytical expressions for the fixation probability of a strategy in a finite population as a function of the strategy’s initial frequency. (DOCX) [file pone.0068153.s002.docx]

**Supporting Information**

**L. Molleman, I. Pen & F.J. Weissing: Effects of conformism on the cultural evolution of social behaviour**

In the first part of this supplement, we provide a graphical illustration of the logistic functions used in our stochastic model to translate information on payoff or frequency differences into the probability of switching to another strategy. In the second part, we derive a diffusion approximation of our stochastic model that yields, by good approximation, analytical expressions for the fixation probability of a strategy in a finite population as a function of the strategy’s initial frequency.

1. **Modelling the probability of switching to another behaviour as a function of payoff or frequency differences**

The stochastic model presented in Section 4 of the main text specifies probabilities of switching as a logistic function of the difference in payoffs or frequencies of strategy *A* and *B* (eqs. 6a,b and 7a,b). Figure S1 below illustrates this relationship between the payoff or frequency differences and switching probabilities for three values of *β*. The function $[1+\exp(-\beta\cdot x)]^{-1}$specifies switching probabilities when two individuals with opposite strategies are paired, given the difference *x* in their payoffs or frequencies. Parameter *β* specifies the relationship between this difference and the probability of switching. When *β* is large, switching is strongly biased towards more frequent behaviour (or behaviours with higher payoffs). When *β* is small, switching is error-prone; a switch towards rare (or lower-payoff) behaviour will frequently occur by conformist (or payoff-based) learning.

**Figure S1. Illustration of the logistic function for three values of *β*.**

1. **Diffusion approximation of our stochastic model**

The stochastic model of the main text tracks the abundance *i* of behaviour *A* in a population of size *n* using transition probabilities between states *i* of the population. In each time step, this state *i* may change into *i*+1 or *i*–1, otherwise, the state remains unchanged. Here we use standard methods [S1, ch 14 and 15; S2] to derive an analytical approximation of this discrete process by means of a diffusion model. The approximation transforms the discrete stochastic model in such a way, that the time and state space intervals become so small that the change of the system can be analyzed as if it were continuous.

For switching probabilities, we take the definition of $T_{i}^{+}$and $T_{i}^{-}$ from the updating process described in section 3 of the Analysis and Results. In the diffusion model, it is convenient to work with the frequency$p=i/n$ of behaviour *A* and the rescaled time $t'=t/n^{2}$. We have to derive the drift coefficient $\mu(p)$ and the diffusion coefficient $\sigma^{2}(p)$ for small time steps ($\Delta t'\to\infty$), which in the chosen parameterization corresponds to large values of *n* ($n\to\infty$).

Using the method in Otto & Day (2007, Box 15.2), it is straightforward to derive the first 3 moments of the expected frequency change per unit rescaled time:

$$m_{1}=\frac{1}{\Delta t'}E\left(\Delta p|p=i/n\right)=n\left(T_{i}^{+}-T_{i}^{-}\right)$$

$$m_{2}=\frac{1}{\Delta t'}E\left(\Delta p^{2}|p=i/n\right)=T_{i}^{+}+T_{i}^{-}$$

$$m_{3}=\frac{1}{\Delta t'}E\left(\Delta p^{3}|p=i/n\right)=n^{-1}\left(T_{i}^{+}-T_{i}^{-}\right)$$

In the limit $n\to\infty$, the third moment vanishes and the drift and diffusion terms are given by, respectively,

$$\begin{matrix} \mu(p)=\lim_{n\to\infty} m_{1}=\frac{1}{2}p(1-p)[pz_{1}+(1-p)z_{2}-(1-2p)z_{3}] \\ \sigma^{2}(p)=\lim_{n\to\infty} m_{2}=p(1-p) \end{matrix}$$

where the terms $z_{k}$ are given by

$$\begin{matrix} z_{1}=(1-\gamma)(a-c)n\beta_{P} \\ z_{2}=(1-\gamma)(b-d)n\beta_{P} \\ z_{3}=\gamma n\beta_{C} \end{matrix}$$

The diffusion approximation is valid if the terms $z_{k}$ remain finite for large values of *n*, i.e. when $n\beta_{P}$ and $n\beta_{C}$ remain bounded. In other words, $\beta_{P}$ and $\beta_{C}$ are of order $\frac{1}{n}$: payoff-based learning and frequency-based learning are both weak in large populations.

With the help of the drift and the diffusion coefficient, we can now express the fixation probability of *A* as a function of the initial frequency of *A*, $p_{0}=k/n$ . In general, this probability is given by the equation (Otto & Day, 2007, p.677):

, with $A(p)=2\int\frac{\mu(p)}{\sigma^{2}(p)}dp.$

Inserting $\mu(p)$ and $\sigma^{2}(p)$ calculated above reveals that can be expressed in terms of the error function (ref):

with

$\rho_{k}=(k/n)(2\cdot(z_{1}-z_{2}+2\cdot z_{3}))+z_{2}-z_{3}$ (S7a)

$$h=\sqrt{2\cdot(z_{1}-z_{2}+2\cdot z_{3})}$$

This approximation of fixation probabilities matches the fixation probabilities in the stochastic model quite well (see Figure 2 of the main text).

**References**

S1. Otto, S. P. and T. Day. (2007). *A Biologist's Guide to Mathematical Modeling in Ecology and Evolution*. Princeton University Press.

S2. Karlin S, Taylor HE. (1975) A first course in stochastic processes. Academic press.
